# Supplementary material for: Synthesis and Characterization of Functionalized Chitosan Nanoparticles with Pyrimidine Derivative for Enhancing Ion Sorption and Application for Removal of Contaminants
Source: Materials (Basel). 2022 Jul 3;15(13):4676. doi: 10.3390/ma15134676 (PMC9267285; doi:10.3390/ma15134676)
Supplement: Supplementary file 1 [file materials-15-04676-s001.zip › materials-1758895-supplementary.pdf]

materials

Article

Synthesis and Characterization of Functionalized Chitosan Nanoparticles with Pyrimidine Derivative for Enhancing Ion Sorption and Application for Removal of Contaminants

Mohammed F. Hamza [1,2,*], Yuezhou Wei [1,3,*], Khalid Althumayri [4], Amr Fouda [5] and Nora A. Hamad [6]

1 School of Nuclear Science and Technology, University of South China, Hengyang 421001, China; m_fouda21@hotmail.com (M.F.H.); yzwei@sjtu.edu.cn (Y.W.)
2 Nuclear Materials Authority, POB 530, El-Maadi, Cairo 11728, Egypt; m_fouda21@hotmail.com (M.F.H.); hamedmira@yahoo.com (H.I.M.)
3 School of Nuclear Science and Engineering, Shanghai Jiao Tong University, Shanghai 200240, China; yzwei@sjtu.edu.cn
4 Department of Chemistry, College of Science, Taibah University, 30002 Al-Madinah Al-Munawarah, Saudi Arabia; Kthumairi@taibahu.edu.sa
5 Botany and Microbiology Department, Faculty of Science, Al-Azhar University, Nasr City, Cairo 11884, Egypt; amr_fh83@azhar.edu.eg
6 Chemistry Department, Faculty of Science, Menofia University, Shebin El-Kom 32511, Egypt; nhamad059@gmail.com
* Correspondence: m_fouda21@hotmail.com (M.F.H.); yzwei@sjtu.edu.cn (Y.W.); Tel.: +20-111-668-1228 (M.F.H.); +86-771-322-4990 (Y.W.)

Citation: Hamza, M.F.; Wei, Y.; Althumayri, K.; Fouda, A.; Hamad, N.A. Synthesis and Characterization of Functionalized Chitosan Nanoparticles with Pyrimidine Derivative for Enhancing Ion Sorption and Application for Removal of Contaminants. *Materials* **2022**, *15*, 4676. https://doi.org/10.3390/ma15134676

Academic Editors: Alexander Yu Churyumov and Federico Mazzucato

Received: 21 May 2022
Accepted: 30 June 2022
Published: 3 July 2022

Publisher's Note: MDPI stays neutral with regard to jurisdictional claims in published maps and institutional affiliations.

Copyright: © 2022 by the authors. Licensee MDPI, Basel, Switzerland. This article is an open access article distributed under the terms and conditions of the Creative Commons Attribution (CC BY) license (https://creativecommons.org/licenses/by/4.0/).

Table S1a. Modeling of uptake kinetics [1-3].

Model	Equation	Parameters	Ref.
PFORE	$q(t) = q_{eq,1}(1 - e^{-k_1 t})$	$q_{eq,2}$ (mmol g^{-1}): sorption capacity at equilibrium<br>k_1 (min^{-1}): apparent rate constant of PFORE	[2]
PSORE	$q(t) = \frac{q_{eq,2}^2 k_2 t}{1 + k_2 q_{eq,2} t}$	$q_{eq,2}$ (mmol g^{-1}): sorption capacity at equilibrium<br>k_2 (g $mmol^{-1}$ min^{-1}): apparent rate constant of PSORE	[2]
RIDE	$\frac{q(t)}{q_{eq}} = 1 - \sum_{n=1}^{\infty} \frac{6\alpha(\alpha+1)\exp\left(\frac{-D_e q_n^2}{r^2} t\right)}{9 + 9\alpha + q_n^2 \alpha^2}$<br>With q_n being the non-zero roots of<br>$\tan q_n = \frac{3\, q_n}{3 + \alpha q_n^2}$ and $\frac{m\, q}{V\, C_0} = \frac{1}{1+\alpha}$	D_e(m^2 min^{-1}) : Effective diffusivity coefficient	[1]

(m (g): mass of sorbent; V (L): volume of solution; C_0 (mmol L^{-1}): initial concentration of the solution).

Table S1b. Modeling of sorption isotherms [4,5].

Model	Equation	Parameters	Ref.
Langmuir	$q_{eq} = \frac{q_{m,L} C_{eq}}{1 + b_L C_{eq}}$	$q_{m,L}$ (mmol g^{-1}): Sorption capacity at saturation of monolayer<br>b_L (L $mmol^{-1}$): Affinity coefficient	[5]
Freundlich	$q_{eq} = k_F C_{eq}^{1/n_F}$	k_F and n_F: empirical parameters of Freundlich equation	[4]
Sips	$q_{eq} = \frac{q_{m,S} b_S C_{eq}^{1/n_S}}{1 + b_S C_{eq}^{1/n_S}}$	$q_{m,L}$, b_S and n_S: empirical parameters of Sips equation (based on Langmuir and Freundlich equations)	[5]

Akaike Information Criterion, AIC:

$$AIC = N \ln\left(\frac{\sum_{i=0}^{N}\left(y_{i,exp.} - y_{i,model}\right)^2}{N}\right) + 2N_p + \frac{2N_p(N_p+1)}{N - N_p - 1}$$

Where N is the number of experimental points, N_p the number of model parameters, $y_{i,exp.}$ and $y_{i,model}$ the experimental and calculated values of the tested variable.

Table S2. Cost evaluations of 10 g MC-TDP sorbent for treatment of Cr(VI) ions.

**Chemicals used**	**Available units and prices**		**Units/prices for 10 g sorbent**	
	**Unit**	**Price (Euro)**	**Unit**	**Price (Euro)**
Synthesis of TDP				
Diethyl malonate	500 g	49	3.75 g	0.367
Thiourea	1000 g	121	1.5 g	0.1815
Sodium ethoxide	500 g	74	50 g	7.4
Synthesis of magnetite				
Ferrous sulfate	250 g	30	4.166 g	0.4999
ammonium ferric (III) sulfate dodecahydrate	500 g	61	6.125 g	0.747
acetic acid	1000 mL	27	9 mL	0.243
Synthesis of modified sorbent				
Chitosan	50 g	72	3 g	4.32
EPI	1000 mL	60	17 mL	1.02
Ethanol	2500 mL	61	50 mL	1.22
DMF	2500 mL	51	140 mL	2.856
Acetone	1000 mL	21	30 mL	0.63
Overall prices for 10 g sorbent				19.4844 euro

Table S3. FTIR characterization of thiourea, TDP, MC-TDP, after sorption and after five cycles of sorption desorption.

**Assignment**	**Thiourea**	**TDP**	**MC-TDP**	**MC-TDP+Cr**	**Sorbent after 5th cycles**	**Ref.**
N-H and OH str.	3375, 3291, 3170	3584, 3392	3443	3423	3398	[6,7]
C-H (aromatic str.)		3095				[8]
C-H (aliphatic str.)	2915, 2850	2867	2912, 2816	2920, 2847	2918, 2872	[9,10]
S-H str. of thiol group	2673	2583	2583		overlapped	[11]
Multiple of bonded C-O			2046		2056	[11]
C=C aromatic str.		1705				[11]
C=O, C=N str. and N-H bend.	1611	1659, 1554	1626, 1499	1633	1628	[12-14]
C-N str., C-H bend, and C-C str.	1467, 1410	1422	1410		1451	[12,13,15-17]
Arom. (secondary amine)/ quaternary ammonium		1338	1363	1380	1370	[11]
Tert. amine		1271	1303		1314	[11]
OH bend (in-plane) and C-O str.,		1243	1214		1235	[7,18,19]
N-C str. and NH_2 (rock.)	1078	1155	1097, 1060	1088	1130	[12,18,20]
C-O str bridge oxygen and C-O-C. str. (asymm)					1056	[21]
NH_2 rock.			1015		1025	[12]
Aromatic C-H bend (in-plane)		923	854		899	[17,21]
NH_2+CO wag. (out-of-phase)	727	800				[11,12]
N-C-N in-plane (bend), and Fe-O	627	599	632	580	627	[15,16,22,23]
O-H out-of-plane (bend.)		541	541		575	[24,25]
C-S str.	485	453				[26,27]
Polysulfide str.	447	448	420		443	[11]

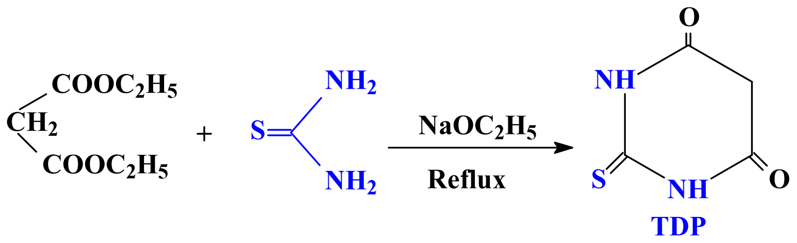

Scheme S1. Synthesis of 2-thioxodihydropyrimidine-4,6(1H,5H)-dione: TDP.

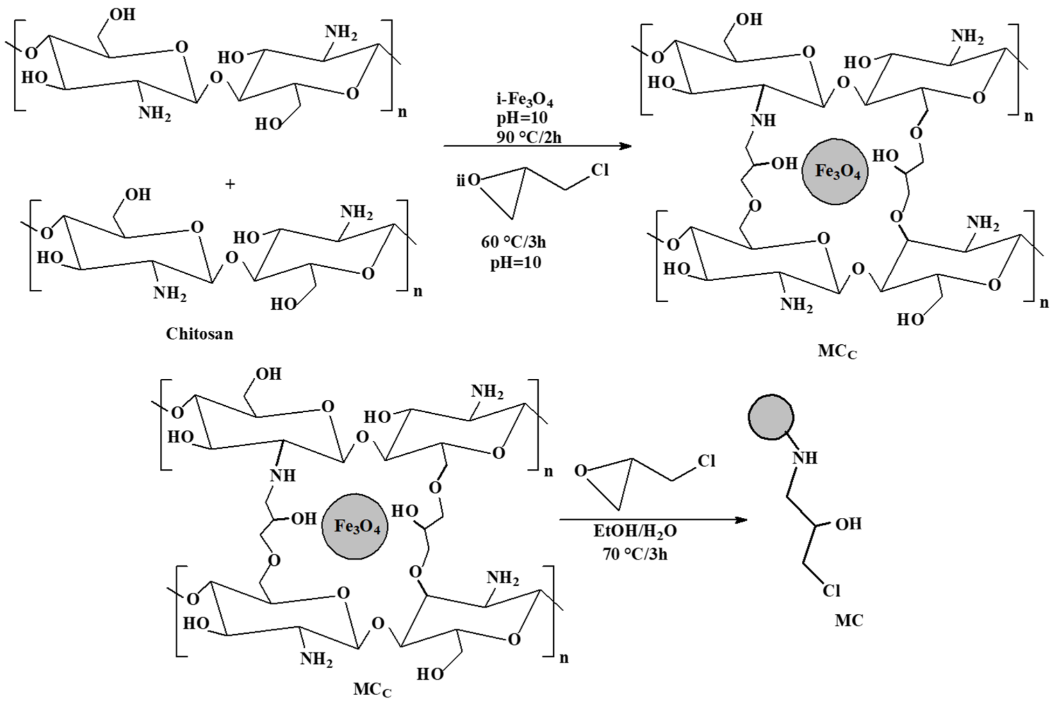

Scheme S2. synthesis of chitosan nanoparticles and activated chitosan (spacer arm).

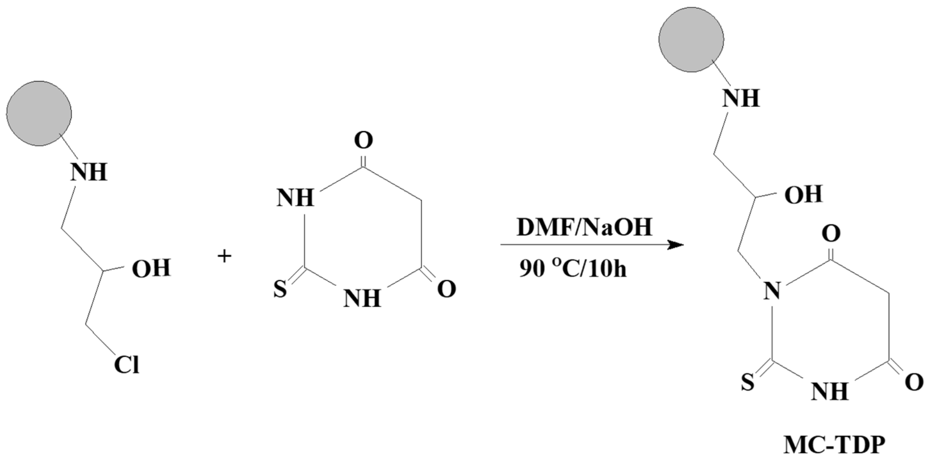

Scheme S3. Synthesis of grafted TDP chitosan nanoparticles.

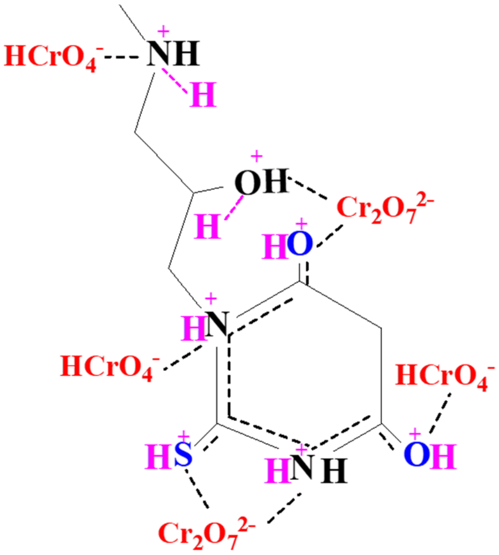

Scheme S4. Expected binding mechanism of chromate ions with MC-TDP sorbent at acidic pH medium.

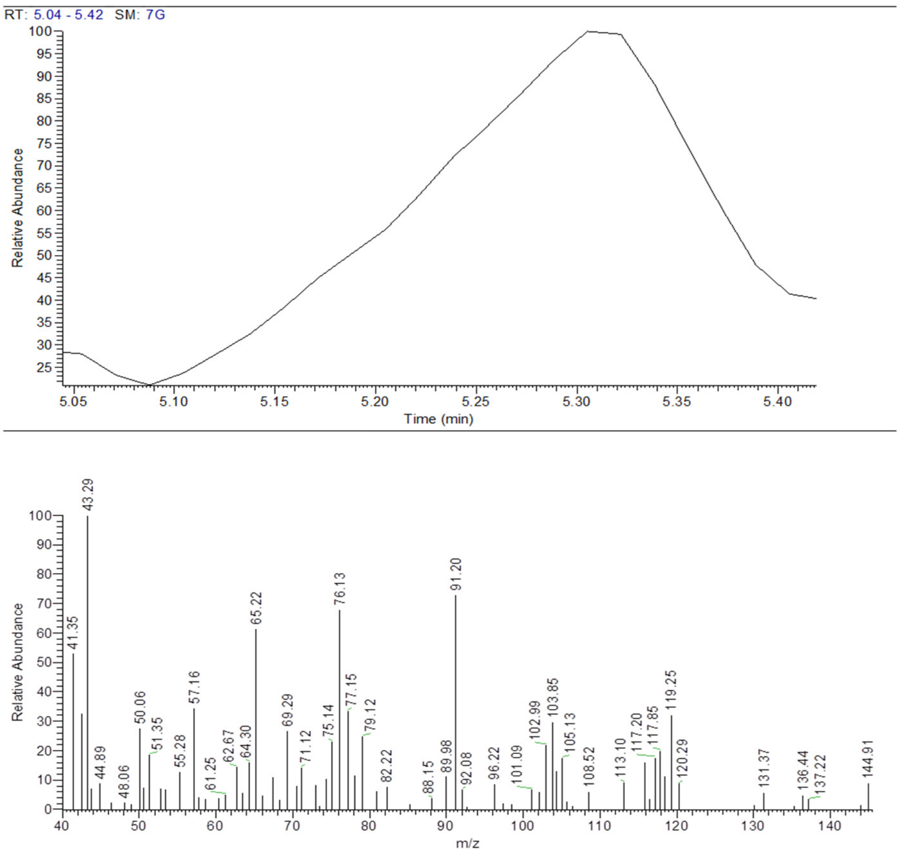

Figure S1. Completed data of the mass spectroscopy for the TDP moiety.

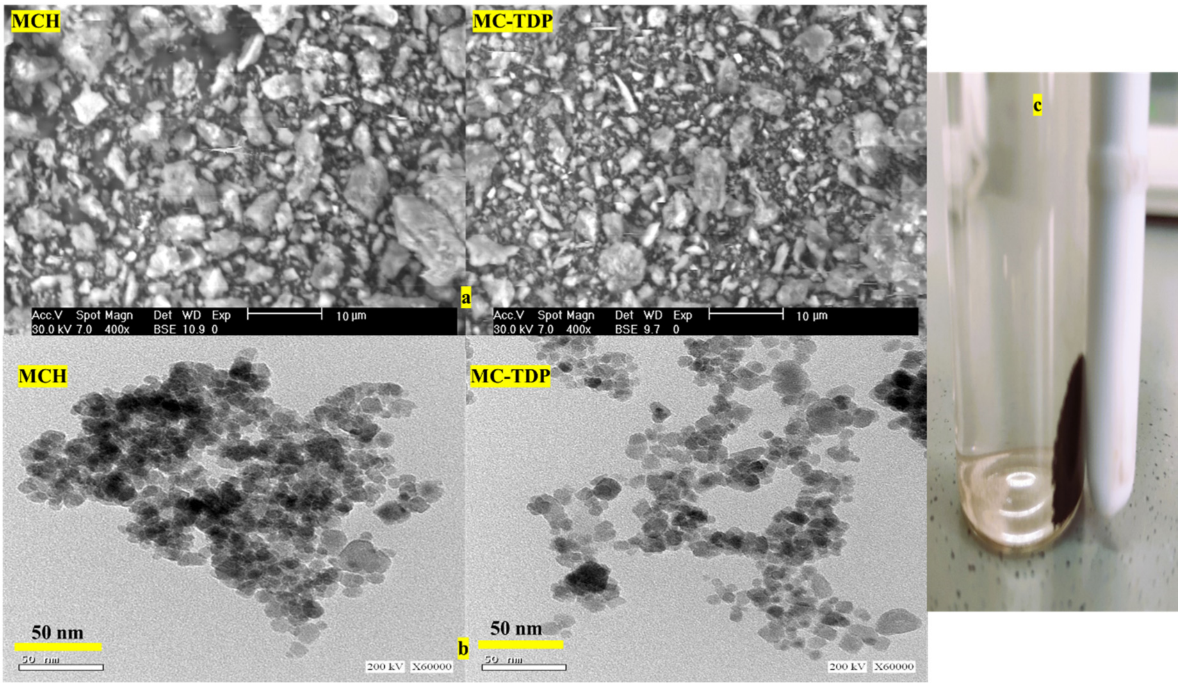

Figure S2. SEM (**a**) and TEM (**b**) graphs of MCH and MC-TDP sorbents and the effect of external magnet on the modified sorbent (**c**).

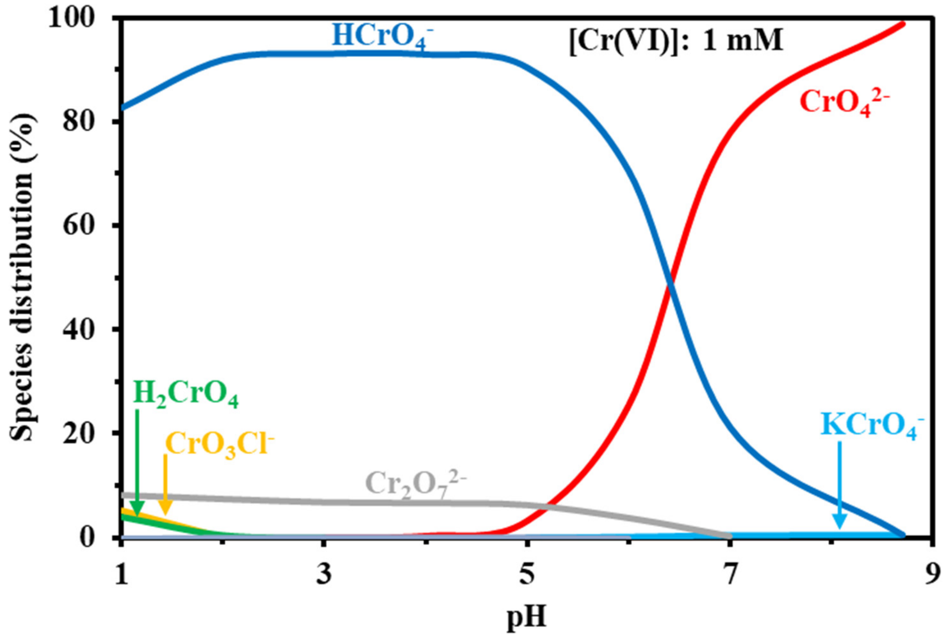

Figure S3. Cr(VI) species at pH 1-9.

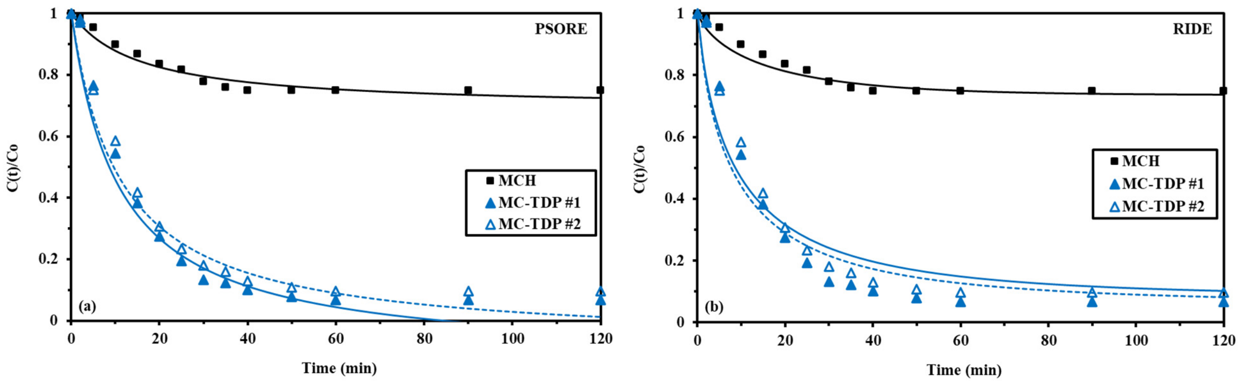

Figure S4. Unfitted uptake Cr(VI) kinetics on MCH, and MC-TDP Modeling with the PSORE (**a**) and RIDE (**b**).

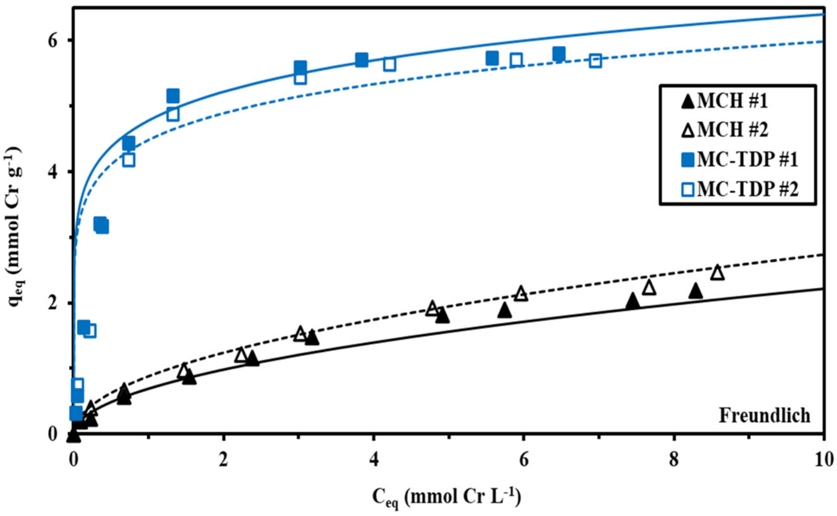

Figure S5. Freundlich modeling equations of sorption isotherms for Cr(VI) using MCH, MC-TDP.

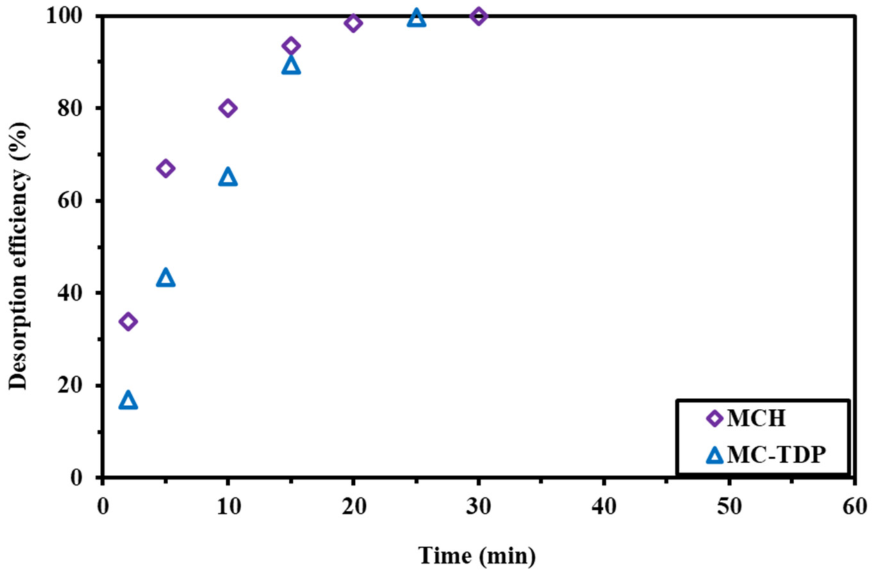

Figure S6. Desorption kinetics of MCH and MC-TDP sorbents.

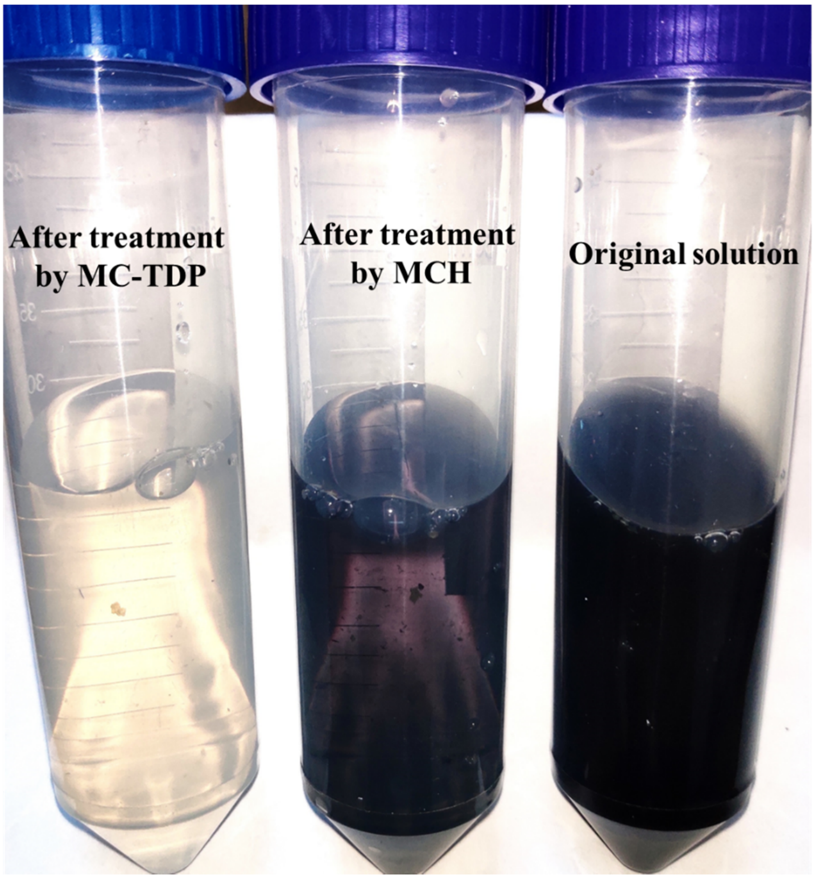

Figure S7. Removal efficient of Cr(VI) using MCH and MC-TDP.

References

1. Crank, J. *The Mathematics of Diffusion*, 2nd. ed.; Oxford University Press: Oxford, U.K., 1975; p. 414.
2. Ho, Y.S.; McKay, G. Pseudo-second order model for sorption processes. *Process Biochem.* **1999**, *34*, 451-465, doi:10.1016/S0032-9592(98)00112-5.
3. Zhang, R.; Leiviska, T. Surface modification of pine bark with quaternary ammonium groups and its use for vanadium removal. *Chem. Eng. J.* **2020**, *385*, Art. N° 123967, doi:10.1016/j.cej.2019.123967.
4. Freundlich, H.M.F. Uber die adsorption in lasungen. *Z. Phys. Chem.* **1906**, *57*, 385-470.
5. Tien, C. *Adsorption Calculations and Modeling*; Butterworth-Heinemann: Newton, MA, 1994 p. 243.
6. Hamza, M.F.; Abdel‐Rahman, A.A.H.; Guibal, E. Magnetic glutamine‐grafted polymer for the sorption of U (VI), Nd (III) and Dy (III). *Journal of Chemical Technology & Biotechnology* **2018**, *93*, 1790-1806.
7. Hamza, M.F. Uranium recovery from concentrated chloride solution produced from direct acid leaching of calcareous shale, Allouga ore materials, southwestern Sinai, Egypt. *Journal of Radioanalytical and Nuclear Chemistry* **2018**, *315*, 613-626.
8. Jin, W.; Shen, D.; Liu, Q.; Xiao, R. Evaluation of the co-pyrolysis of lignin with plastic polymers by TG-FTIR and Py-GC/MS. *Polymer degradation and stability* **2016**, *133*, 65-74.
9. Hamza, M.F.; Mahfouz, M.G.; Abdel-Rahman, A.A.-H. Adsorption of uranium (VI) ions on hydrazinyl amine and 1, 3, 4-thiadiazol-2 (3 H)-thion chelating resins. *Journal of dispersion science and technology* **2012**, *33*, 1544-1551.
10. Hamza, M.F.; Wei, Y.; Guibal, E. Quaternization of algal/PEI beads (a new sorbent): Characterization and application to scandium sorption from aqueous solutions. *Chemical Engineering Journal* **2020**, *383*, 123210.
11. Coates, J. Interpretation of Infrared Spectra, A Practical Approach. In *Encyclopedia of Analytical Chemistry* John Wiley & Sons, Ltd.: 2006; pp. 1-23.
12. Piasek, Z.; Urbanski, T. The infra-red absorption spectrum and structure of urea. *Bull. Acad. Pol. Sci.* **1962**, *X*, 113-120.
13. Manivannan, M.; Rajendran, S. Investigation of inhibitive action of urea-Zn^{2+} system in the corrosion control of carbon steel in sea water. *Int. J. Environ. Sci. Technol.* **2011**, *3*, 8048-8060.
14. Wei, Y.; Salih, K.A.; Rabie, K.; Elwakeel, K.Z.; Zayed, Y.E.; Hamza, M.F.; Guibal, E. Development of phosphoryl-functionalized algal-PEI beads for the sorption of Nd (III) and Mo (VI) from aqueous solutions–Application for rare earth recovery from acid leachates. *Chemical Engineering Journal* **2021**, *412*, 127399.
15. Chandra, S.; Saleem, H.; Sundaraganesan, N.; Sebastian, S. Experimental and theoretical vibrational spectroscopic and HOMO, LUMO studies of 1,3-dimethylbarbituric acid. *Ind. J. Chem.* **2009**, *48A*, 1219-1227.
16. Corazzari, I.; Nistico, R.; Turci, F.; Faga, M.G.; Franzoso, F.; Tabasso, S.; Magnacca, G. Advanced physico-chemical characterization of chitosan by means of TGA coupled on-line with FTIR and GCMS: Thermal degradation and water adsorption capacity. *Polym. Degrad. Stabil.* **2015**, *112*, 1-9, doi:10.1016/j.polymdegradstab.2014.12.006.
17. Lawrie, G.; Keen, I.; Drew, B.; Chandler-Temple, A.; Rintoul, L.; Fredericks, P.; Grondahl, L. Interactions between alginate and chitosan biopolymers characterized using FTIR and XPS. *Biomacromolecules* **2007**, *8*, 2533-2541, doi:10.1021/bm070014y.

18. Yao, Z.; Zhang, C.; Ping, Q.; Yu, L.L. A series of novel chitosan derivatives: synthesis, characterization and micellar solubilization of paclitaxel. *Carbohydrate Polymers* **2007**, *68*, 781-792.
19. Wei, Y.; Salih, K.A.; Lu, S.; Hamza, M.F.; Fujita, T.; Vincent, T.; Guibal, E. Amidoxime functionalization of algal/polyethyleneimine beads for the sorption of Sr (II) from aqueous solutions. *Molecules* **2019**, *24*, 3893.
20. Tsai, H.S.; Wang, Y.Z.; Lin, J.J.; Lien, W.F. Preparation and properties of sulfopropyl chitosan derivatives with various sulfonation degree. *J. Appl. Polym. Sci.* **2010**, *116*, 1686-1693, doi:10.1002/app.31689.
21. Duarte, M.L.; Ferreira, M.C.; Marvao, M.R.; Rocha, J. An optimised method to determine the degree of acetylation of chitin and chitosan by FTIR spectroscopy. *Int. J. Biol. Macromol.* **2002**, *31*, 1-8, doi:10.1016/s0141-8130(02)00039-9.
22. Hamza, M.F.; Abdel-Rahman, A.A.-H.; Negm, A.S.; Hamad, D.M.; Khalafalla, M.S.; Fouda, A.; Wei, Y.; Amer, H.H.; Alotaibi, S.H.; Goda, A.E.-S. Grafting of Thiazole Derivative on Chitosan Magnetite Nanoparticles for Cadmium Removal—Application for Groundwater Treatment. *Polymers* **2022**, *14*, 1240.
23. Hamza, M.F.; Wei, Y.; Benettayeb, A.; Wang, X.; Guibal, E. Efficient removal of uranium, cadmium and mercury from aqueous solutions using grafted hydrazide-micro-magnetite chitosan derivative. *Journal of Materials Science* **2020**, *55*, 4193-4212.
24. Wei, Y.; Salih, K.A.; Hamza, M.F.; Fujita, T.; Rodríguez-Castellón, E.; Guibal, E. Synthesis of a New Phosphonate-Based Sorbent and Characterization of Its Interactions with Lanthanum (III) and Terbium (III). *Polymers* **2021**, *13*, 1513.
25. Wei, Y.; Rakhatkyzy, M.; Salih, K.A.; Wang, K.; Hamza, M.F.; Guibal, E. Controlled bi-functionalization of silica microbeads through grafting of amidoxime/methacrylic acid for Sr (II) enhanced sorption. *Chemical Engineering Journal* **2020**, *402*, 125220.
26. Caetano, C.S.; Caiado, M.; Farinha, J.; Fonseca, I.M.; Ramos, A.M.; Vital, J.; Castanheiro, J.E. Esterification of free fatty acids over chitosan with sulfonic acid groups. *Chem. Eng. J.* **2013**, *230*, 567-572, doi:10.1016/j.cej.2013.06.050.
27. Xiang, Y.; Yang, M.; Guo, Z.B.; Cui, Z. Alternatively chitosan sulfate blending membrane as methanol-blocking polymer electrolyte membrane for direct methanol fuel cell. *J. Membr. Sci.* **2009**, *337*, 318-323, doi:10.1016/j.memsci.2009.04.006.
